# Effect of family history of diabetes and obesity status on lifetime risk of type 2 diabetes in the Iranian population

**DOI:** 10.7189/jogh.12.04068

**Published:** 2022-08-09

**Authors:** Azra Ramezankhani, Ali Siamak Habibi-Moeini, Seyed Saeed Tamehri Zadeh, Fereidoun Azizi, Farzad Hadaegh

**Affiliations:** 1 Prevention of Metabolic Disorders Research Center, Research Institute for Endocrine Sciences, Shahid Beheshti University of Medical Sciences, Tehran, Iran; 2 Endocrine Research Center, Research Institute for Endocrine Sciences, Shahid Beheshti University of Medical Sciences, Tehran, Iran

## Abstract

**Background:**

Data are scarce for the lifetime risk of diabetes in the Middle East and North Africa region countries. We estimated the lifetime risk of type 2 diabetes among Iranian adults at age 20 and 40 years, and their variation by family history of diabetes and body mass index (BMI).

**Methods:**

The data from 8435 diabetes-free participants from the Tehran Lipid and Glucose study were used in this analysis. We estimated the lifetime risk of diabetes stratified by sex, and quantified the impact of family history of diabetes and BMI status on the lifetime risks, singly and jointly.

**Results:**

At age 20 years, the overall lifetime risk of diabetes was 57.8% (95% CI = 54.0%-61.8%) for men and 61.3% (57.2%-65.4%) for women. Having both family history of diabetes and increased level of BMI, alone, increased the lifetime risk of diabetes in both sexes. Moreover, the simultaneous presence of family history of diabetes and overweigh/obesity increased the lifetime risk of diabetes in both sexes. So that, at age 20 years the lifetime risk in obese men with positive family history of diabetes was about 54% higher, compared to normal weight men without family history of diabetes; the corresponding value for women was 42%. Also, normal weight men without family history of diabetes lived 24 years longer free of diabetes, compared with obese men with family history of diabetes. In women, the corresponding value was 20 years.

**Conclusions:**

Our study shows the alarming lifetime risk of diabetes across the strata of BMI, which emphasizes the need for more effective interventions to reduce incidence, particularly, among individuals with a positive family history of diabetes.

Diabetes mellitus is a major global health problem worldwide with the global prevalence of 463 million people in 2019; this number is expected to be about 700 million by 2045. Currently, the Middle East and North Africa (MENA) region has the highest prevalence of diabetes and is projected to have second highest global growth in terms of projections of diabetes increase by 2030 [1]. Among Iranian adults, the prevalence of diabetes was 11.9% in 2011 and this number is projected to be nearly 9.2 million in the year 2030 [2].

Although incidence and prevalence estimates provide useful information for the overall burden of diabetes in a community and at a particular time, but do not provide sufficient information regarding the future risk of diabetes at the individual level. Lifetime risk estimates provide the cumulative risk of a disease over a person’s remaining lifetime, beginning at a particular age. This estimate provides straightforward messages about the risk of a certain disease during the life span, and it is therefore preferable to use in clinical practice [3,4].

A few studies from some of high income countries [5-9] have reported the lifetime risk of diabetes. Accordingly, the lifetime risk at 40 years of age ranged from 21% to 23% in women and from 17% to 26% in men. Moreover, in India, as a low- to middle income country, the lifetime risk of diabetes was 64.6 and 55.5% in women and men aged ≥20 years, respectively [10]. However, there are no estimates of lifetime risk of diabetes in MENA region countries. Hence, to address this gap in the literature and given the higher prevalence of diabetes in this region, we estimated the residual lifetime risk of diabetes among Iranian population. As diabetes is caused by both genetic and environmental risk factors, we further estimated the role of body mass index (BMI), as a most important diabetes risk factor in Iran [11], and family history of diabetes (FHD) singly and jointly on the lifetime risk of diabetes stratified by sex. We hypothesized that higher levels of BMI and positive FHD, each singly  and in combination, were associated with higher lifetime risk for diabetes.

## METHODS

### Study population

**Figure 1:**
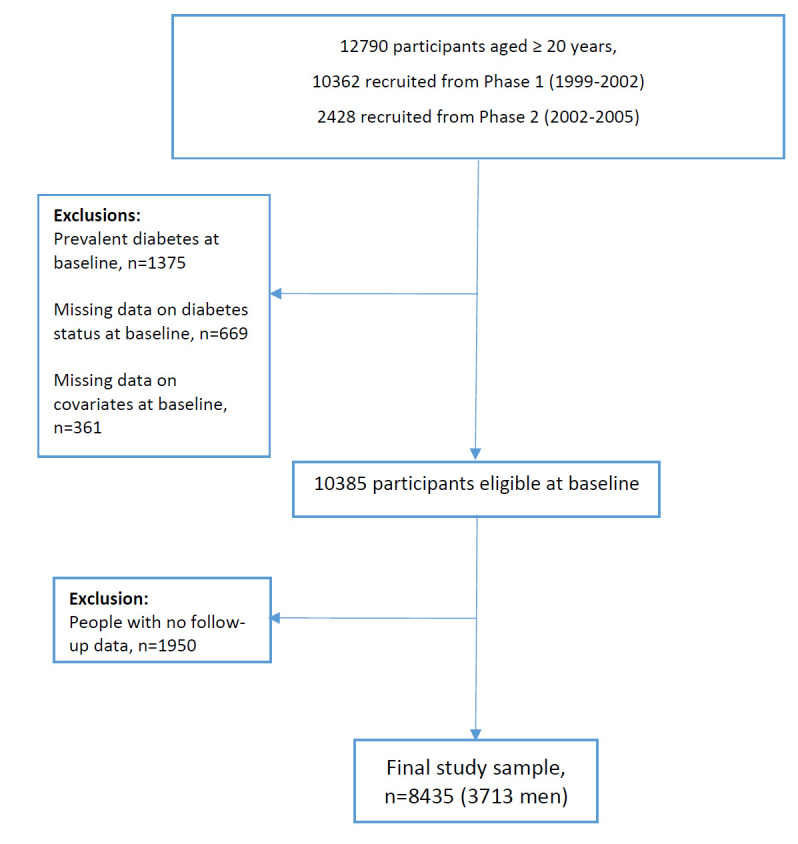
Flowchart of sample selection for the study.

This study is embedded within the framework of the Tehran Lipid and Glucose Study (TLGS), a population-based prospective cohort study of non-communicable diseases and related risk factors among the residents living in Tehran, capital of Iran. The study design of the TLGS has been described in detail previously [12]. Briefly, in 1999, a total of 15 005 individuals aged ≥3 years who were selected using multistage cluster random sampling method participated in the study. In 2002, a population of 3550 individuals was enrolled, bringing the total study population to 18 555 individuals. All participants have been assessed in intervals of three years with medical histories, physical examinations and laboratory tests. Until now, six examinations (phase) 1 (1999-2001), 2 (2002-2005), 3 (2005-2008), 4 (2009-2011), 5 (2012-2015), and 6 (2015-2018) have been conducted. Besides the triennial reexaminations, all participants have been followed annually for any medical outcome leading to hospitalization and death by telephone call to them or their family [13]. For the present study, 12 790 participants aged ≥20 years from the first (n = 10 362) and second (n = 2428) phases were selected as baseline. From this population, we excluded individuals who had diabetes at baseline (n = 1375), people who had missing data on diabetes status at baseline (n = 669) and other covariates (n = 361), and individuals with no follow-up data after recruitment (n = 1950) until the end of the study (April 18, 2018). Finally, 8435 adults (3713 men) remained for our analysis (**Figure 1**).

### Outcome measure

Participants were followed up for the occurrence of diabetes from the date of baseline until the end of the study. At baseline and during follow-up, diagnosis of diabetes was based on fasting plasma glucose (FPG)≥7 mmol/L or 2-hour postload plasma glucose (2-hPG)≥11.1 mmol/L or treatment with glucose lowering medications. Death was determined from the annual follow-up data.

### Assessment of risk factors

Details of the TLGS study design and protocol for assessment of demographic information, anthropometric and biochemical measurements, medical and family histories, and use of drugs at baseline and follow-ups has been previously published [14]. BMI was calculated as body weight (in kg) divided by the square of height (in meters) (kg/m^2^) and were categorized as normal (BMI<25), overweight (BMI≥25 and <30) and obesity (BMI≥30). A FHD was defined as having diabetes in first-degree relatives. Smoking behaviour was categorized into three groups: current smoker (a person who smokes cigarettes or uses other tobacco products daily or occasionally); past-smoker (a formerly daily or occasional smoker who currently does not smoke) and non-smokers (people who never smoked before). Systolic and diastolic blood pressure (SBP, DBP) were measured as the mean of two measurements, taken on the right arm at an interval of five minutes, after subjects had rested 15 minutes before the first measurement, and hypertension was defined as a SBP≥140 mm Hg or a DBP≥90 mm Hg or taking antihypertensive medications [15]. Blood samples were collected from participants after an overnight fast of 12-14 hours to assess FPG, 2-hPG, total cholesterol (TC), triglyceride (TG) and high-density lipoprotein cholesterol (HDL-C).

### Statistical analysis

Baseline characteristics of participants are summarized using descriptive statistics. The normality of distribution was checked for all continuous variables by Kolmogorov-Smirnov (KS) test. For normally distributed continuous variables, mean and standard deviation (SD) are reported, and in the case of non-normally distribution, continuous variables are reported as median and Interquartile (IQ). The categorical variables are presented as frequency and percentages. We also compared baseline characteristics between participants and nonparticipants. Non-participants included those with missing data on diabetes status and covariates at baseline and individuals without any follow-up data. A two-sample *t* test was used for comparison of normally distributed continuous variables and the Mann-Whitney U test for non-normally distributed continuous variables. The χ^2^ test was used for comparison of categorical variables.

All participants were followed from their study entry to the occurrence of the diabetes (outcome of interest), death (competing risk), lost to follow-up or the end of the study period (censored).

In traditional survival analytic techniques, subjects who die during the observation period are often treated as censored observations, and one minus the survival function (the complement of the survival function, F(t) = 1-S(t)), is defined as the cumulative incidence of the event over the observation period [16] which results in biased estimates of incidence [17]. To overcome the issue of such competing risks, the cumulative incidence function (CIF), known as the lifetime risk, is used which allows for estimation of probability of the occurrence of the event over time while taking competing risk into account [16]. In fact, the function CIF denotes the probability of experiencing the event of interest before the occurrence of a competing event [16].

We estimated the remaining lifetime risks for diabetes using a modified version of Kaplan-Meier analysis with survival age as time scale, which accounts for left truncation and right censoring, and the competing risk of death free of diabetes [7,8]. Lifetime risk estimates are competing-risk-adjusted cumulative incidences from a particular age until the participant’s age at last follow-up. In this study, the maximum age was 97.7 years for both men and women at last follow-up. Lifetime risks were calculated at baseline ages 20 and 40 years (referred to as “index” ages) both for the overall participants and for men and women, separately. Additionally, we calculated 10- and 20-year risks for diabetes, at index ages 20 and 40 years. Participants were then stratified into three categories of BMI (<25 kg/m^2^, ≥25 to <30 kg/m^2^, ≥30 kg/m^2^) and two categories of FHD (yes, no), and the lifetime risk of diabetes was examined within each category. Lifetime risks were also estimated in BMI and FHD strata (6 categories). To test the hypothesis of a significant difference in lifetime risks across these strata, the estimated lifetime risks were compared between different categories using a two-sided z-tests [18]. We performed a power analysis (sample size, probability, type 1 and type 2 error rates) to examine the power of the statistical test.

Further, to study the impact of BMI and FHD on onset of diabetes, we examined differences among different strata of these two risk factors in mean of diabetes free survival. Because the censoring generally precludes estimation of the mean survival time, the estimation of these mean survival times was obtained using Irwin’s restricted mean [19], which is the mean of the survival time restricted to a given time point. The choice of the time point was set as 90 years old, because few participants survived after age 90 years [20]. Statistical analysis were performed using IBM SPSS Statistics version 20 (IBM Corp) and R version 4.0.3 with the ‘etm’ and ‘survival’ packages, and a P value <0.05 was set for 2-sided significance test.

## RESULTS

### Baseline characteristics

Baseline characteristics of the participants stratified by sex and index ages are presented in **Table 1**. As TG had skewed distribution, it is shown as median (IQ). For overall study population, the mean (SD) age at baseline was 40.5 (13.8) years, and 4722 (55%) of participants were women. Of the 8435 participants at baseline, 2166 (25.7%) had FHD, 1466 (17.4%) had hypertension, and 1332 (15.8%) were current smokers. The mean of BMI and HDL-C were higher in women than men. However, mean of SBP and median of TG were higher in men than women.

**Table 1:** Baseline characteristics of participants by sex and index ages, Tehran Lipid and Glucose Study (1999-2018)*

		Index age 20 years (n = 8435)		Index age 40 years (n = 3937)	
	**Total population n = 8435**	**Men (n = 3713)**	**Women (n = 4722)**	**Men (n = 1849)**	**Women (n = 2088)**
Age (years)	40.5 (13.8)	42.1 (14.6)	39.4 (13.1)	54.1 (10.3)	51.7 (8.8)
BMI (kg/m^2^)	26.5 (4.6)	25.6 (4.1)	27.2 (4.9)	26.0 (3.8)	29.1 (4.4)
SBP (mmHg)	116.8 (17.3)	118.7 (16.7)	115.4 (17.6)	123.6 (19.5)	124.4 (20.1)
DBP (mmHg)	76.5 (10.6)	76.9 (10.7)	76.2 (10.5)	78.8 (11.5)	80.1 (11.0)
FPG (mmol/L)	4.9 (0.5)	5.0 (0.5)	4.9 (0.5)	5.1 (0.5)	5.1 (0.6)
2 h-PLPG (mmol/L)	5.9 (1.6)	5.7 (1.7)	6.0 (1.5)	6.1 (1.8)	6.5 (1.6)
TC (mmol/L)	5.2 (1.2)	5.2 (1.1)	5.3 (1.2)	5.4 (1.1)	5.9 (1.2)
TG (mmol/L)	1.5 (1.2)	1.7 (1.3)	1.4 (1.1)	1.8 (1.3)	1.8 (1.2)
HDL-C (mmol/L)	1.1 (0.2)	1.0 (0.2)	1.2 (0.3)	1.0 (0.2)	1.2 (0.3)
FHD (yes)	2166 (25.7)	897 (24.2)	1269 (26.9)	372 (20.1)	561 (26.9)
**Smoking**					
Never	6515 (77.2)	2060 (55.5)	4455 (94.3)	968 (52.4)	1936 (92.7)
Past	588 (7.0)	514 (13.8)	74 (1.6)	342 (18.5)	56 (2.7)
Current	1332 (15.8)	1139 (30.7)	193 (4.1)	539 (29.2)	96 (4.6)
Hypertension (yes)	1466 (17.4)	647 (17.4)	819 (17.3)	511 (27.6)	695 (33.3)
Use of lipid lowering drugs (yes)	184 (2.2)	60 (1.6)	124 (2.6)	46 (2.5)	113 (5.4)
Use of blood pressure lowering drugs (yes)	457 (5.7)	139 (3.7)	318 (6.7)	130 (7.0)	295 (14.1)

BMI – body mass index, SBP – systolic blood pressure, DBP – diastolic blood pressure, FPG – fasting plasma glucose, 2h-PLPG-2 – postload plasma glucose, TC – total cholesterol, TG – triglyceride, HDL-C – high-density lipoprotein cholesterol, FHD – family history of diabetes

*Values are presented as mean (SD) for continuous variables, and frequency (%) for categorical variables. For TG, values are shown as median (interquartile range).

Baseline characteristics of participants and non-participants are shown in **Table 2**. Compared with non-participants, participants had lower levels of FPG and HDL-C, and were less likely to smoke and to have hypertension; but, were more likely to have FHD at baseline.

**Table 2:** Baseline characteristics of participants and non-participants, Tehran Lipid and Glucose Study (1999-2018)*

	Participants, n = 8435	Non-participants, n = 2980	*P*-value
Age (years)	40.5 (13.8)	40.1 (15.9)	0.142
BMI (kg/m^2^)	26.5 (4.6)	26.3 (5.1)	0.092
SBP (mmHg)	116.8 (17.3)	117.4 (18.8)	0.186
DBP (mmHg)	76.5 (10.6)	76.5 (10.8)	0.944
FPG (mmol/L)†	4.9 (0.52)	4.9 (0.6)	0.023
2 h-PLPG (mmol/L)	5.9 (1.6)	5.9 (1.6)	0.593
TC (mmol/L)	5.2 (1.2)	5.2 (2.5)	0.240
TG (mmol/L)‡	1.5 (1.2)	1.5 (1.1)	0.164
HDL-C (mmol/L)§	1.1 (0.2)	1.1 (0.2)	0.036
FHD (yes)	2166 (25.7)	687 (23.1)	0.004
**Smoking:**			
Never	6515 (77.2)	1995 (73.4)‖	
Past	588 (7.0)	178 (6.5)	<0.001
Current	1332 (15.8)	546 (20.1)	
Hypertension (yes)	1466 (17.4)	514 (19.1)	0.042
Use of lipid lowering drugs (yes)	184 (2.2)	69 (2.3)	0.668
Use of blood pressure lowering drugs (yes)	457 (5.4)	183 (6.1)	0.150
		

BMI – body mass index, SBP – systolic blood pressure, DBP – diastolic blood pressure, FPG – fasting plasma glucose, 2h-PLPG – 2 postload plasma glucose, TC – total cholesterol, TG – triglyceride, HDL-C – high-density lipoprotein cholesterol, FHD – family history of diabetes

*Values are presented as mean (SD) for continuous variables, and frequency (%) for categorical variables. Values for TG are presented as median (IQ).

†The exact values are 4.96 (0.52) and 4.99 (0.56) for participant and non-participants, respectively.

‡The TG was tested using the Mann-Whitney U test, because of its skewed distribution.

§The exact values are 1.08 (0.28) and 1.09 (0.29) for participant and non-participants, respectively.

‖Non-respondents had 261 missing values on smoking status.

### Risk estimates for diabetes

**Figure 2:**
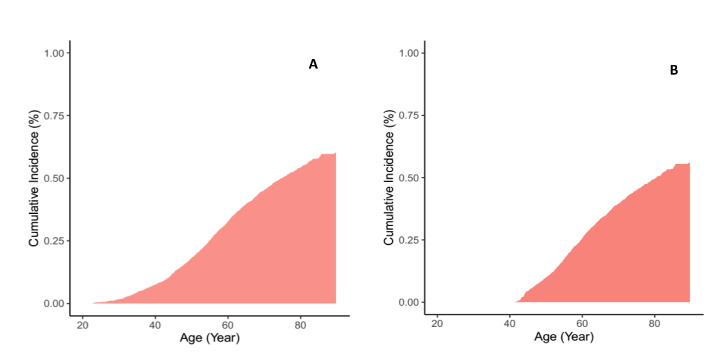
Lifetime risk of diabetes adjusted for the competing risk of death: **Panel A.** At age 20 years. **Panel B.** At age 40 years.

During 106 996 person-years of follow-up, with median (Interquartile range (IQR)) of 15.3 (12.0-16.5) years, a total of 1373 individuals (598 men) developed diabetes and 283 (187 men) died. Among total population, the remaining lifetime, 10-year and 20-year risk for a 20-year-old non-diabetic individual to develop diabetes was 60.2%, 1.6% and 7.4%, respectively (**Table 3**). The corresponding values for a 40-year-old individual were 56.1%, 9.9% and 25.7%. The lifetime and short time risks were slightly higher in women than men in both index ages 20 and 40 years. **Figure 2** shows the cumulative incidence risk in index ages 20 and 40 years among total population.

**Table 3:** Remaining lifetime, 10- and 20-year risks of diabetes, by sex, Tehran Lipid and Glucose Study (1999-2018)

	Risk of diabetes (95% CI)		
**Index age 20 years**	**Total population** **n = 8435**	**Men** **n = 3713**	**Women** **n = 4722**
Lifetime	60.2% (57.3-63.1)	57.8% (54.0-61.8)	61.3% (57.2-65.4)
10-years	1.6% (1.1-2.5)	1.3% (0.6-2.7)	1.8% (1.0-3.1)
20-years	7.4% (6.5-8.6)	6.6% (5.2-8.3)	8.0% (6.7-9.6)
**Index age 40 years**	**n = 3937**	**n = 1849**	**n = 2088**
Lifetime	56.1% (52.8-59.4)	52.9% (48.4-57.3)	57.9% (53.4-62.5)
10-years	9.9% (8.2-11.9)	8.3% (6.1-11.2)	11.1% (8.8-14.1)
20-years	25.7% (23.7-28.0)	22.8% (19.9-26.1)	28.1% (25.2-31.1)

CI – confidence interval

**Figure 3:**
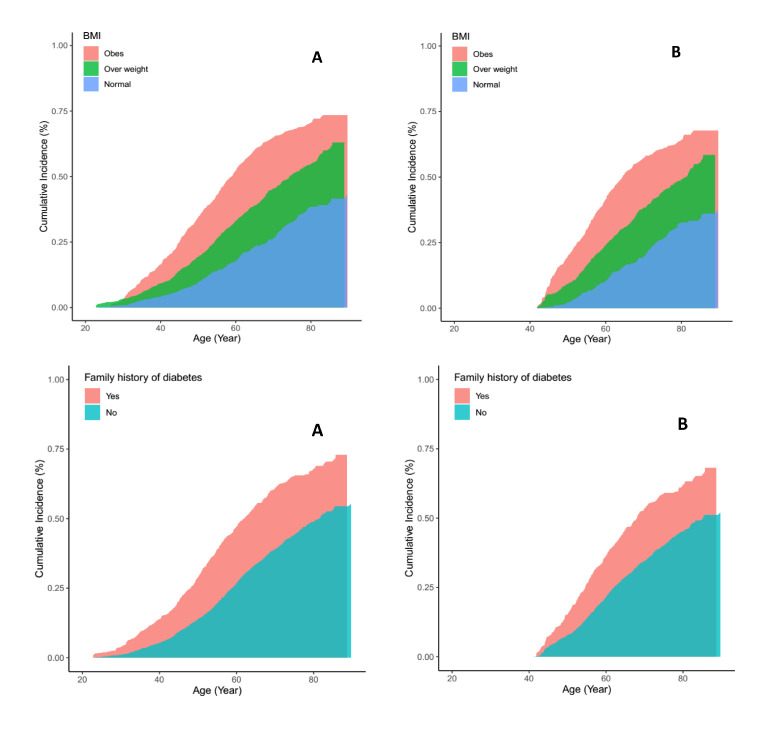
Lifetime risk of diabetes, adjusted for the competing risk of death, by categories of body mass index (BMI) and family history of diabetes: **Panel A.** At age 20 years. **Panel B.** At age 40 years.

With advancing age, lifetime risks of diabetes decreased, however, 10- and 20-year risks increased in both men and women. The increased short time risk of diabetes with advancing age was larger for women than for men; for example, at age 40, the 10-year risk of diabetes was 8.3% in men and 11.1% in women; the corresponding values were 22.8% and 28.1% for 20-year risk of diabetes (**Table 3**). In total population, individuals with normal BMI (<25 kg/m^2^), at both ages 20 and 40 years, had a significantly lower diabetes lifetime risk compared with overweight and obese individuals (**Table 4**). Moreover, in both index ages 20 and 40 years, individuals with FHD had about 17% increased lifetime risk of diabetes, compared with individuals without FHD. In **Figure 3**, we have presented the lifetime risk in different categories of BMI and FHD at aged 20 and 40 years in total population. Lifetime risks in BMI and FHD strata showed that having FHD increased lifetime risks in all categories of BMI; for example, obese individuals with positive FHD had about 19% higher lifetime risk, compared with obese people without FHD at both index ages 20 and 40 years (**Table 4**). The Similar pattern was observed in men and women at age 20 years. In both sexes, the lifetime risk of developing diabetes increased with increasing BMI, and the highest lifetime risk was observed among obese individuals with positive FHD (92.2% and 84.3% in men and women, respectively) (**Table 5**). The power level of the statistical tests in **Table 4** and **Table 5** were in the high range from 86% to 99%.

**Table 4:** Lifetime risk of diabetes at ages 20 and 40 years, by BMI and FHD*

	n	Lifetime risk at age 20 y	*P*-value		n	Lifetime risk at age 40 y	*P*-value
**BMI (kg/m^2^)**							
<25	3246	43.1% (37.5-49.2)	….		1092	37.7% (31.7-44.5)	….
25-30	3405	63.0% (58.4-67.5)	<0.001		1760	58.4% (53.2-63.6)	<0.001
≥30	1784	73.4% (69.4-77.3)	<0.001		1085	67.7% (62.8-72.5)	0.005
**FHD**							
No	6269	55.2% (51.7-58.7)	….		3004	51.9% (48.1-55.8)	….
Yes	2166	72.9% (67.7-77.9)	<0.001		933	68.1% (61.8-74.3)	<0.001
**Combined FHD and BMI**							
BMI<25 without FHD	2526	40.2% (33.8-47.4)	…		879	35.3% (28.6-43.1)	…..
BMI<25 with FHD	720	53.1% (42.4-64.5)	0.027		213	47.6% (35.6-61.2)	0.052
BMI 25-30 without FHD	2487	57.9% (52.5-63.4)	….		1338	55.1% (49.2-61.1)	….
BMI 25-30 with FHD	918	73.7% (65.8-81.0)	<0.001		422	66.5% (56.6-75.9)	0.026
BMI≥30 without FHD	1256	67.1% (62.1-72.1)	…..		787	62.0% (56.3-67.7)	…..
BMI≥30 with FHD	528	86.1% (79.5-91.3)	<0.001		298	80.7% (71.7-88.3)	<0.001

BMI – body mass index, FHD – family history of diabetes

*Life time risks were estimated from age of 20 y. Life time risks were compared using z-test, and *P*-values are for the comparison with the preceding category (eg, BMI≥30 with FHD with BMI≥30 without FHD).

**Table 5:** Lifetime risk at age of 20 years for diabetes in men and women, by BMI and FHD

	Lifetime risk of diabetes			Lifetime risk of diabetes		
	**N**	**Men (n = 3713)**	***P*-value**	**n**	**Women (n = 4722)**	***P-*value**
**BMI (kg/m^2^)**						
<25	1625	41.5% (35.1-48.7)	….	1621	46.6% (37.4-56.8)	….
25-30	1580	63.9% (58.1-69.7)	<0.001	1825	60.0% (52.9-67.4)	0.015
≥30	508	74.7% (68.1-80.9)	0.007	1276	73.2% (68.3-77.9)	0.002
**FHD**						
No	2816	52.6% (48.1-57.4)	….	3453	55.8% (51.2-60.6)	….
Yes	897	71.4% (64.1-78.3)	<0.001	1269	73.7% (66.5-80.4)	<0.001
**Combined FHD and BMI**						
BMI<25 without FHD	1277	38.0% (30.7-46.3)	…	1249	42.0% (33.3-53.8)	…
BMI<25 with FHD	348	51.8% (40.9-63.6)	0.026	372	61.1% (38.4-84.1)	0.089
BMI 25-30 without FHD	1182	59.5% (52.6-66.5)	…	1305	54.2% (46.1-62.8)	…
BMI 25-30 with FHD	395	71.6% (60.3-82.0)	0.035	520	73.1% (61.6-83.4)	0.004
BMI≥30 without FHD	357	69.1% (60.6-77.1)	….	899	67.1% (61.2-73.0)	….
BMI≥30 with FHD	151	92.2% (84.7-96.8)	<0.001	377	84.3% (76.5-90.7)	<0.001

BMI – body mass index, FHD – family history of diabetes

**Figure 4:**
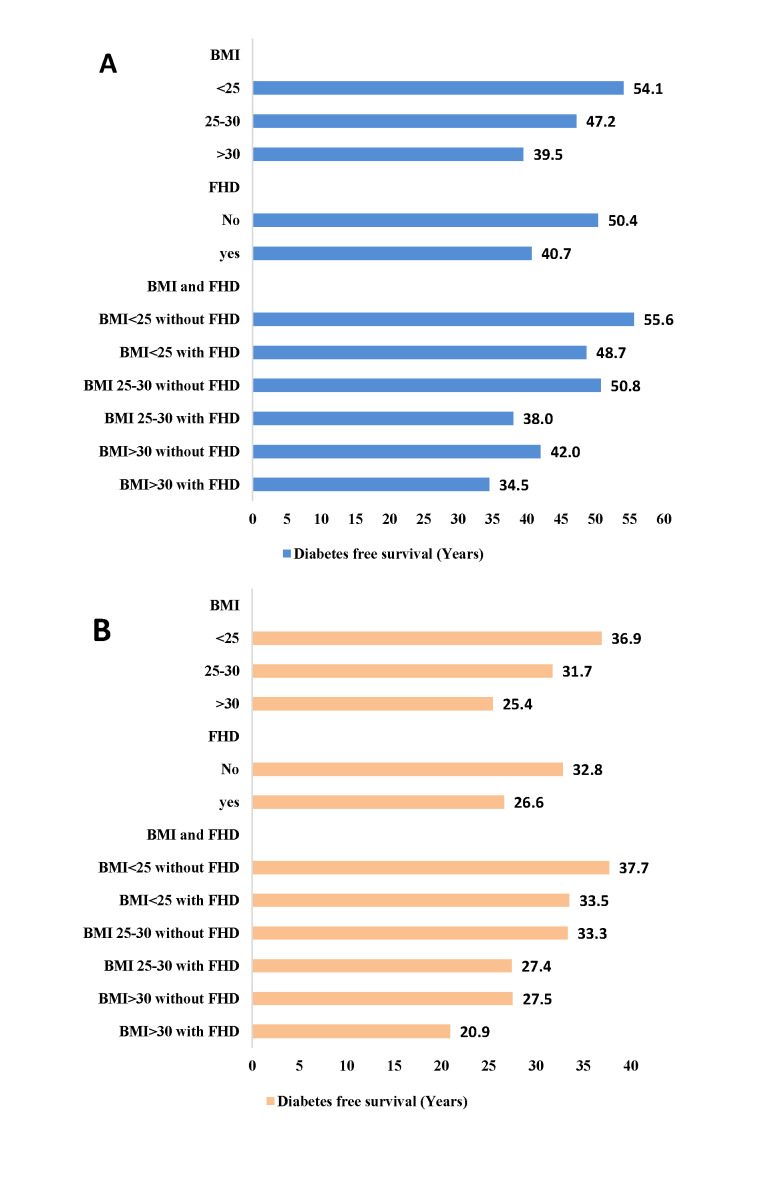
Survival free of diabetes (years) by body mass index and family history of diabetes: **Panel A.** After the age of 20. **Panel B.** After the age of 40 years.

**Figure 5:**
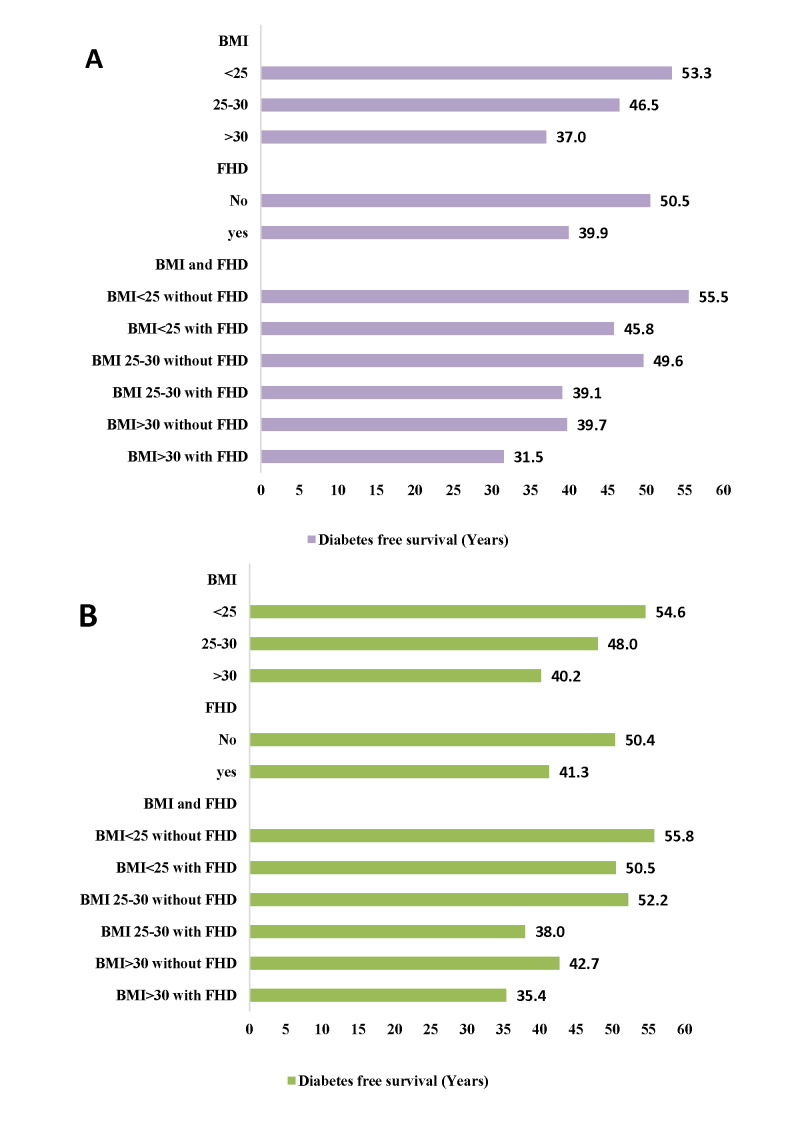
Survival free of diabetes (years) after the age of 20 years by body mass index and family history of diabetes: **Panel A.** Among men. **Panel B**. Among women.

Remaining life-years free of diabetes, from age 20 and 40 years in total population by BMI and FHD strata are shown in the **Figure 4**. In both index age of 20 and 40 years, longer survival free of diabetes was observed among individuals with normal BMI (<25 kg/m^2^) and without FHD. For example, at an index age of 20 years, individuals with normal BMI and without FHD lived up to 19 years longer free of diabetes than obese individuals with positive FHD. Overall, years lived without diabetes decreased with increasing BMI. Also, positive FHD decreased years lived without diabetes in all BMI strata; as shown, at index age 20, years lived without diabetes were 8 years higher in obese individuals without FHD, compared with obese people with FHD. Additionally, **Figure 4** shows that at age 20, the average age of onset of diabetes in individuals with normal BMI and without FHD was more than 19 years later compared with those with BMI>30 kg/m^2^ and positive FHD. Similarly, at age 20 years, both normal weight men and women without FHD expect to live about 55 years of their remaining life years free of diabetes. However, the diabetes free survival were slightly higher in women than men, show a delay in the onset of diabetes among women than men (**Figure 5**).

## DISCUSSION

For the first time, in this population-based cohort study from MENA region, we showed that about 60% of non-diabetic individuals develop diabetes from the age of 20 years onwards. In short-term (10-year and 20-year), the overall risks of diabetes were 1.6% and 7.4%, respectively. The overall lifetime risk was decreased with increasing age; while, the short-term risks were increased. Both the overall life time and short-term risks were generally equal in women and men. Furthermore, we found that being overweight/obese or having positive FHD affects the risk of developing diabetes and decreases the number of years lived without diabetes. Additionally, the simultaneous presence of these risk factors (obesity and positive FHD) was associated with 46% higher lifetime risk, and living 19 years less free of diabetes, compared with the absence of these two risk factors, at age of 20 years.

Lifetime risk of diabetes in our population was higher than U.S [21] Brazilian [22] and Dutch [7] populations, which confirms the report of International Diabetes Federation that MENA region has the highest worldwide diabetes prevalence [1]. Also, lifetime risk of diabetes at both index ages 20 and 40 years was almost equal in both men and women. Two study in India and Canada documented higher lifetime risk of diabetes at age 20 years in women than men [10]. In contrast, an Australian [6] and American [21] study reported the higher lifetime risk of diabetes in men. According to above mentioned studies, diversity is existed among different ethnicities in terms of sex-specific diabetes incidence, which can be explained by different factors including, but not limited to, biology, environment, lifestyle, and socioeconomic status [23].

We found a substantial effect of obesity on lifetime risk of diabetes in both genders, which is in agreement with previous findings. For example, a study among U.S population showed that at age of 18 years, the absolute lifetime risk of diabetes were 9.9%, 37.2%, and 50.5% higher in over-weight, obese, and very obese men, compared to normal-weight men. The corresponding values for women were 18.3%, 37.5%, and 57.3% [5]. In other study from India, lifetime risk of diabetes at age of 20 years was highest among obese individuals: 86.0% and 86.9% in women and men, respectively [10]. In our study, the impact of overweight and obesity on lifetime risk at age of 20 was higher than US, but lower than Indian population [10] and first nation in Canada [9].

We also found the higher lifetime risk of diabetes among individuals with positive FHD, compared with individuals without FHD, as expected [24-26]. Moreover, we found that among individuals with positive FHD, those with obesity had about 33% higher lifetime risk of diabetes, compared to normal weight individuals. This finding is in line with the previous studies which showed that preventing overweight/obesity through lifestyle intervention attenuates high genetic risk [27]. A very recent study assessed whether genetic predisposition can influence the association between cardiovascular health (CVH) and lifetime risk of type 2 diabetes among middle-aged individuals and found that favourable CVH was associated with lower remaining lifetime risk of diabetes regardless of genetic predisposition [28]. Notably, since the trend of obesity is increasing in Iran [29,30] and obesity is the main cause of many health issues, there is an urgent need to implement preventive strategies to reduce obesity.

As far as we aware, no study has investigated the effect of FHD on lifetime risk of diabetes. A very recent study estimated the lifetime risk of diabetes in two cohorts (Atherosclerosis Risk in Communities (ARIC) study and the Rotterdam Study (RS)), based on genetic susceptibility. They revealed that individuals aged 45 years with high genetic risk score had almost 14% higher lifetime risk of diabetes compared to those with low genetic risk score [8]. As individuals with genetic risk factors will carry the risk of the disease throughout their life, they prefer to know the absolute risks of diabetes from birth through the end of life. From a clinical perspective, because FHD in part reflects unmeasured genetic determinants of the diabetes [31], thus, information on family history can help identify individuals with high prevalence of genetic risk at early stages, who will take advantage from intensified lifestyle intervention.

Of note, even though at both index ages of 20 and 40 years in our study, about 43% and 37% of individuals with normal weight eventually developed diabetes, their age at onset of diabetes were about 15 and 10 years later than individuals with obesity. Thus, the maintenance of optimal weight through ages 20 and 40 years may not guarantee a life free from diabetes, but, it increases the number of years lived free of diabetes. The high prevalence of obesity among Iranian adults [11,29], necessitating a proactive policy that prevents obesity among Iranian population.

### Strengths and limitations

The current study has some strength that worth to be mentioned. First, this is a prospective population-based cohort study with a two decade of follow-up in MENA region, which allowed us to estimate the lifetime risk of developing diabetes precisely. Second, in the present study, diabetes diagnosis was based on FPG, 2-hPG and using blood glucose-lowering medications. Some previous studies applied self-reported diabetes status for definition of diabetes status, which can be one of the main reasons behind differences in findings of some relevant studies. For example, two studies conducted among U.S population estimated the lifetime risk of diabetes based on self-reported data [5,21], and since there was a high prevalence of undiagnosed diabetes in U.S (over 25%) [32] there was a good possibility that their estimations were underestimated [33,34]. Third, in order to reduce the risk of overestimation, we estimated the lifetime risk of diabetes with adjustment for the competing risk of death.

Our study is also subjects to several limitations. The main limitation of the current study is a 26% of loss to follow-up. The statistically, but not clinically important differences were observed between the participants and non-participants in some baseline variables; the participants had slightly higher levels of FPG and were more likely to have a FHD (25% vs 23%) compared with non-participants, but they had higher levels of HDL-c and were less likely to smoke. Moreover, there were no significant differences between two groups in other risk factors of diabetes. On the other hand, the incidence rate of diabetes in our study is very similar to other Iranian cohorts [35,36]. Thus, lost to follow-up does not seem to have a major effect on the estimated risk of diabetes in our analysis. The second limitation is that our study population was selected from adult residents of Tehran; hence, our findings cannot be generalized to other rural areas of the Iran.

## CONCLUSIONS

For the first time, we demonstrated that over half of the adult residents of Metropolitan Tehran are at risk of developing diabetes throughout their lives. Highest lifetime risk of diabetes in individuals with high BMI and positive FHD, and also, lower diabetes-free survival among them highlights the importance of developing an effective strategy to prevent obesity and implementing diabetes screening and prevention programme in our country.
